# Supplementary material for: Comprehensive Analysis of GABAA-A1R Developmental Alterations in Rett Syndrome: Setting the Focus for Therapeutic Targets in the Time Frame of the Disease
Source: Int J Mol Sci. 2020 Jan 14;21(2):518. doi: 10.3390/ijms21020518 (PMC7014188; doi:10.3390/ijms21020518)
Supplement: Supplementary file 1 [file ijms-21-00518-s001.pdf]

International Journal of *Molecular Sciences*

MDPI

SUPPLEMENTARY MATERIAL

Comprehensive Analysis of $GABA_A$-A1R Developmental Alterations in Rett Syndrome: Setting the Focus for Therapeutic Targets in the Time Frame of the Disease

Alfonso Oyarzabal [1,*], Clara Xiol [2,†], Alba Aina Castells [2,†], Cristina Grau [1], Mar O'Callaghan [3], Guerau Fernndez [2], Soledad Alcántara [4], Mercè Pineda [3], Judith Armstrong [2], Xavier Altafaj [5,‡], and Angels García-Cazorla [1,‡]

International Journal of *Molecular Sciences*
MDPI

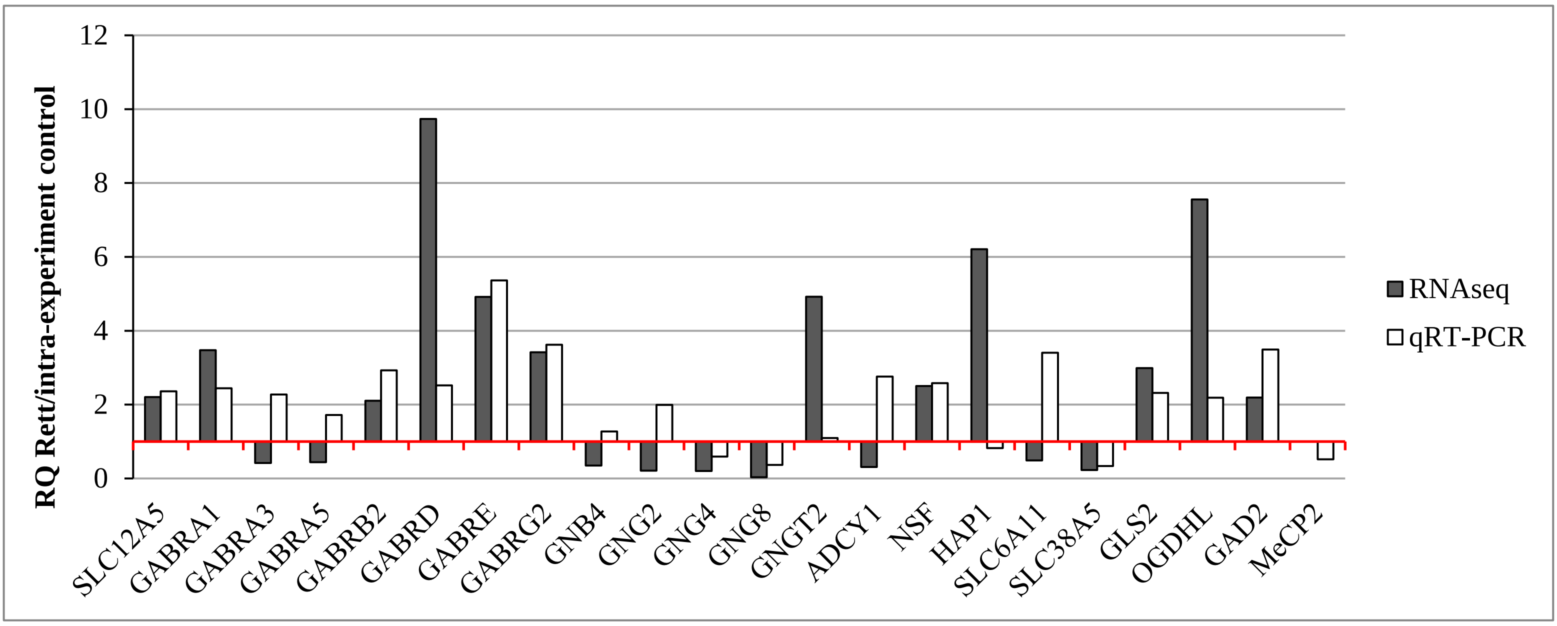

Supplementary Figure 1: RNAseq experiment validation through qPCR: The qRT-PCR measurements showed that 20 out of the 21 genes called as differentially expressed in the RNAseq experiment were also called as differentially expressed in the qRT-PCR experiment, thus validating the results.
